# Biepicondylar fracture presenting with elbow dislocation: a case report

**DOI:** 10.1186/1752-1947-6-265

**Published:** 2012-08-31

**Authors:** Savas Guner, Sukriye Ilkay Guner, Mehmet Fethi Ceylan, Gokay Gormeli, Cemile Ayse Gormeli, Haci Onder

**Affiliations:** 1 Department of Trauma and Orthopedic Surgery, Medical School of Yuzuncu Yil University, Van, Turkey; 2 School of Nursing, Yuzuncu Yil University, Van, Turkey; 3 Department of Trauma and Orthopedic Surgery, Van Training and Research Hospital, Van, Turkey; 4 Department of Radiology, Van Training and Research Hospital, Van, Turkey

## Abstract

**Introduction:**

Biepicondylar fracture of the elbow is very rare, and to date there have only been three reports of this injury and its treatment in the English scientific literature. This case report evaluates the surgical internal fixation of a biepicondylar fracture of the elbow with an associated dislocation.

**Case presentation:**

We report the case of a 15-year-old Turkish girl with a biepicondylar fracture dislocation of the left elbow. Open reduction and an internal fixation operation were applied. There were no complications.

**Conclusion:**

In these injuries, open reduction and internal fixation appear to be a good method to restore elbow stability and function.

## Introduction

Elbow fractures are quite common in the pediatric age group [1]. In a study of 400 consecutive elbow fractures in children, medial epicondylar fracture was the third most common fracture after supracondylar and lateral condylar fractures [2]. However, biepicondylar fracture dislocation of the elbow is very rare, and to date there have only been three reports of this injury and its treatment in the English scientific literature [2-4].

In this case report, we evaluate the results of the surgical treatment of a biepicondylar fracture of the elbow with an associated dislocation by internal fixation.

## Case presentation

A 15-year-old Turkish girl came to our hospital after falling onto her outstretched left arm five days earlier. Before coming to our department, she was seen by another medical institution and had been put in a posterior splint with no reduction maneuver being attempted. Our patient reported severe pain in her elbow and a sensation that her left elbow was ‘out of place’.

**Figure 1:**
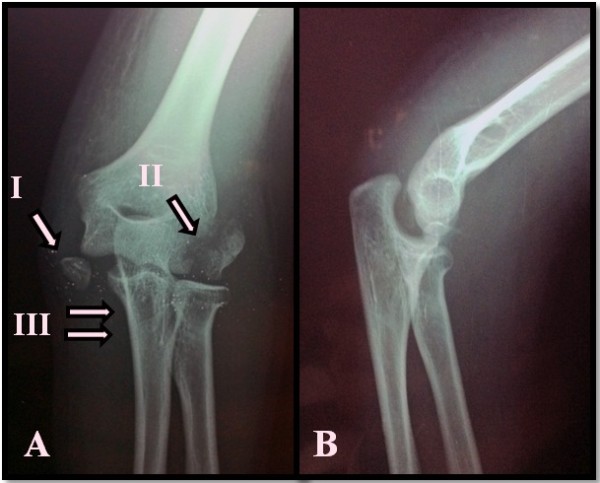
**X-ray views of the left elbow.** (**A**) Anteroposterior X-ray view of the left elbow (I: medial epicondyle avulsion fracture, II: lateral epicondyle displace fracture, III: humero-ulnar joint dislocation); (**B**) Lateral X-ray view of the left elbow shows widening of the joint space.

**Figure 2:**
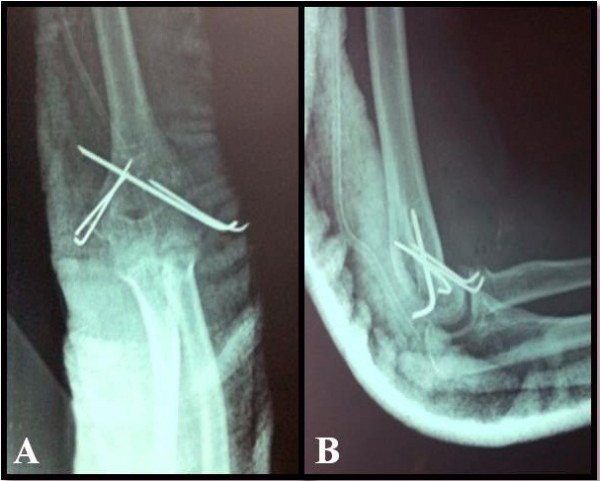
**Postoperative anteroposterior and lateral X-ray views of the left elbow**.

On physical examination, our patient’s elbow was mildly swollen and tender to palpation over the lateral and medial aspect. The range of motion of her elbow was limited due to the pain and the elbow itself was grossly unstable. Her left upper extremity was neurologically intact. The elbow joint was not obviously dislocated but radiography showed the joint to be nonconcentric and subluxated (Figure 1). There was an avulsion fracture of her medial epicondyle and a lateral humeral epicondyle. The fracture of the lateral epicondyle of her humerus was extended into the capitellum. Our patient was taken to the operating room. After the elbow dislocation was reduced under general anesthesia, an open reduction and internal fixation was applied to the biepicondylar fracture (Figure 2). No complications occurred during the surgery. Our patient’s arm was splinted in a cast for four weeks and a nurse explained to our patient how to care for the cast. After removing the cast, our patient was recommended to undertake range of motion exercises.

## Discussion

Fractures of the medial epicondyle are commonly caused by a valgus stress producing traction on the flexor-pronator tendon and subsequently on the medial epicondyle itself. The valgus stress may be produced by a fall on the outstretched hand or by a fall on the elbow. Direct trauma is a less common cause of medial epicondylar injury [1]. Medial stability of the elbow depends on the forearm flexors and the medial collateral ligament. When the medial epicondyle is displaced, the collateral ligament is completely relocated with the fracture part of the medial epicondyle. When the tightness of the medial collateral ligament decreases, medial instability of the elbow is likely to occur. Therefore, surgical treatment is recommended for patients with a displaced fracture of the medial epicondyle [5].

Isolated lateral epicondyle fractures are not commonly reported. Lateral epicondyle fracture is frequently caused by a serious varus force applied to the elbow and can occur from a direct blow or avulsion forces from the extensor muscles [2,6]. A reasonable explanation for the mechanism of biepicondylar fractures is a fall on outstretched hand, in which there is valgus stress at the elbow together with internal rotation of the humerus over the planted forearm and hand, which leads to traction and avulsion forces on both epicondyles [2]. Biepicondylar elbow fracture dislocation can cause gross instability of the elbow [2]. If instability occurs, surgical reduction and fixation of the epicondyle is an effective method of treatment [7].

## Conclusions

In the medical literature, there is limited data available describing biepicondylar fracture dislocation of the elbow in children [2-4]. In these injuries, open reduction and internal fixation appear to be a good method to restore elbow stability and function. Orthopedic surgeons should also bear in mind dislocations in pediatric fractures in joint areas.

## Consent

Written informed consent was obtained from the patient’s legal guardian for publication of this case report and accompanying images. A copy of the written consent is available for review by the Editor-in-Chief of this journal.

## Competing interests

The authors declare that they have no competing interests.

## Authors’ contributions

SG was the main author and performed the clinical assessment, surgery and follow-up. HO performed the clinical assessment and the follow-up. GG performed the bibliographic research. MFC performed the clinical assessment and the surgery. SIG was a major contributor in writing the manuscript and performed the cast care. CAG performed the radiological assessment. All authors have read and approved the final manuscript.
